# Trends in Incidence of Type 1 and Type 2 Diabetes Among Youths — Selected Counties and Indian Reservations, United States, 2002–2015

**DOI:** 10.15585/mmwr.mm6906a3

**Published:** 2020-02-14

**Authors:** Jasmin Divers, Elizabeth J. Mayer-Davis, Jean M. Lawrence, Scott Isom, Dana Dabelea, Lawrence Dolan, Giuseppina Imperatore, Santica Marcovina, David J Pettitt, Catherine Pihoker, Richard F. Hamman, Sharon Saydah, Lynne E. Wagenknecht

**Affiliations:** ^1^Division of Health Services Research, Department of Foundations of Medicine, New York University Long Island School of Medicine, Mineola, New York; ^2^Departments of Nutrition and Medicine, University of North Carolina, Chapel Hill, North Carolina; ^3^Department of Research & Evaluation, Kaiser Permanente Southern California, Pasadena, California; ^4^Department of Biostatistics and Data Science, Wake Forest School of Medicine, Winston-Salem, North Carolina; ^5^Department of Epidemiology, Colorado School of Public Health, Aurora, Colorado; ^6^Department of Endocrinology, Children's Hospital Medical Center, Cincinnati, Ohio; ^7^Division of Diabetes Translation, National Center for Chronic Disease Prevention and Health Promotion, CDC; ^8^Northwest Lipid Research Laboratory, Seattle Washington; ^9^Kaiser Permanente Southern California, Pasadena, California; Santa Barbara, California; ^10^Department of Pediatrics, University of Washington, Seattle, Washington; ^11^Department of Epidemiology, Colorado School of Public Health, University of Colorado Denver, Aurora, Colorado; ^12^Division of Public Health Sciences, Wake Forest School of Medicine, Winston-Salem, North Carolina.

Diabetes is one of the most common chronic diseases among persons aged <20 years (*1*). Onset of diabetes in childhood and adolescence is associated with numerous complications, including diabetic kidney disease, retinopathy, and peripheral neuropathy, and has a substantial impact on public health resources (*2*,*3*). From 2002 to 2012, type 1 and type 2 diabetes incidence increased 1.4% and 7.1%, respectively, among U.S. youths (*4*). To assess recent trends in incidence of diabetes in youths (defined for this report as persons aged <20 years), researchers analyzed 2002–2015 data from the SEARCH for Diabetes in Youth Study (SEARCH), a U.S. population-based registry study with clinical sites located in five states. The incidence of both type 1 and type 2 diabetes in U.S. youths continued to rise at constant rates throughout this period. Among all youths, the incidence of type 1 diabetes increased from 19.5 per 100,000 in 2002–2003 to 22.3 in 2014–2015 (annual percent change [APC] = 1.9%). Among persons aged 10–19 years, type 2 diabetes incidence increased from 9.0 per 100,000 in 2002–2003 to 13.8 in 2014–2015 (APC = 4.8%). For both type 1 and type 2 diabetes, the rates of increase were generally higher among racial/ethnic minority populations than those among whites. These findings highlight the need for continued surveillance for diabetes among youths to monitor overall and group-specific trends, identify factors driving these trends, and inform health care planning.

SEARCH is a population-based registry of diabetes with surveillance of 69,457,475 youths (aged <20 years) covering geographically defined populations in Colorado (all 64 counties plus selected Indian reservations in Arizona and New Mexico under the direction of Colorado), Ohio (eight counties), South Carolina (all 46 counties), Washington (five counties), and Kaiser Permanente Southern California (KPSC) health plan enrollees in seven counties (*3*). Although the SEARCH population is similar demographically to the U.S. youth population (*4*), it is not designed to be nationally representative. Case reports were obtained from medical records and validated based on physician diagnosis of diabetes. Eligible participants included nonmilitary and noninstitutionalized persons with diabetes diagnosed at age <20 years, who resided in one of the study counties at the time of diagnosis; for persons in California eligibility required membership in KPSC and for American Indians, participation in Indian Health Services at the time of diagnosis (*3*,*4*). Race and ethnicity were based on self-report (82%), medical records (15%), or geocoding (3%). Diabetes type was noted as the physician-assigned type at 6 months after diagnosis. Incidence rates are reported for all type 1 diabetes in persons aged <20 years. Because the number of type 2 diabetes cases diagnosed in children aged <10 years were too few to report trends in this age group (181 total cases during 2002–2015), incident cases of type 2 diabetes are only included for persons aged 10–19 years at diagnosis.

For each incident year, the annual denominators included all civilian residents of the SEARCH sites in the same age ranges on December 31 of that year (*3*,*4*). Incidence rates and 95% confidence intervals (CIs) are presented as 2-year moving averages and expressed per 100,000 person-years (*5*). A change point [or joinpoint] was placed at the year 2011 based on an information criteria measure (*6*). Comparisons were made between the periods 2002–2010 and 2011–2015 to determine whether the annual percentage change (APC) was constant over the 2002–2015 period. Consistency of the incidence trends over time by age, sex, and race/ethnicity was evaluated by testing for interaction between each of these variables separately with the change point at year 2011 using segmented regression. Rates adjusted for age, sex, and race/ethnicity and estimation of the change in the annual incidence trends during 2002–2015 are reported. A statistically significant change in incidence trends is indicated when the 95% CI excluded zero. Incidence trends were modeled separately for type 1 and type 2 diabetes assuming a negative binomial distribution with a logarithmic link and using a generalized autoregressive moving average to account for serial correlation and presented by race/ethnicity (*7*). Completeness of case ascertainment for the four geographically based centers was assessed using capture/recapture, where the number of times an individual case was found, either in hospital or other clinical setting, was used to estimate the number of missed cases (*8*). SAS (version 9.4; SAS Institute) and R (version 3.5.2; The R Foundation) statistical software were used for analyses.

During 2002–2015, among 69,457,475 youths at risk for diabetes, SEARCH identified 14,638 youths with type 1 diabetes and 3,916 with type 2 diabetes. Based on the capture/recapture analysis, few cases were missed, with 98%–99% completeness of ascertainment of cases of type 1 and 92%–97% for type 2 diabetes.

Incidence of type 1 diabetes increased during 2002–2015 in all demographic groups except those who received a diagnosis at age <5 years and American Indians (Figure) (Table 1). Incidence of type 1 diabetes differed by age at diagnosis, sex, and race/ethnicity, with higher rates observed among persons aged 10–14 years, males, and whites. The overall APC adjusted for age, sex, and race/ethnicity in type 1 diabetes incidence was 1.9% per year over the entire period (2002–2015). The APC remained constant for children and adolescents aged 5–19 years, in males, and in females. Steeper increases in age-adjusted and sex adjusted incidence of type 1 diabetes were seen among blacks (2.7% per year), Hispanics (4.0% per year) and Asians and Pacific Islanders (4.4% per year) than among whites (0.7% per year). Incidence among Asians and Pacific Islanders did not change significantly during 2002–2010, then steeply increased during 2011–2015 (8.5% per year).

**FIGURE:**
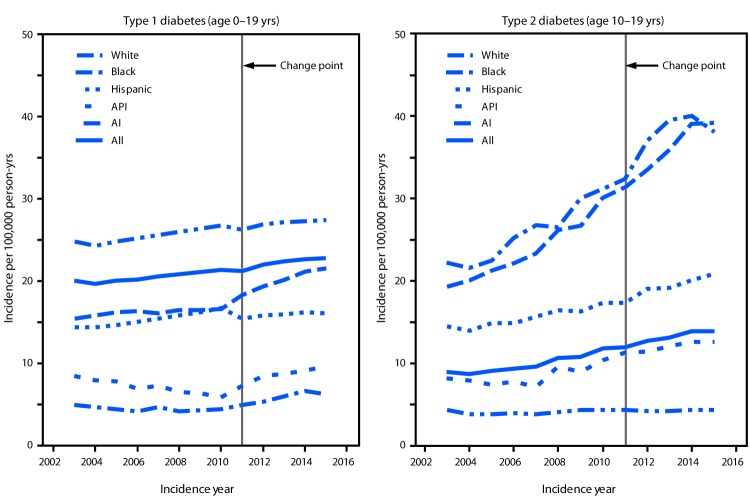
Model-adjusted incidence of type 1 and type 2 diabetes among youths, overall and by race/ethnicity* — SEARCH for Diabetes in Youth Study (SEARCH), United States,^†^ 2002–2015 **Abbreviations:** AI = American Indian; API = Asian/Pacific Islander. * Persons who were AI were primarily from one southwestern tribe. ^†^ SEARCH includes data on youths (<20 years) in Colorado (all 64 counties plus selected Indian reservations in Arizona and New Mexico under the direction of Colorado), Ohio (eight counties), South Carolina (all 46 counties), Washington (five counties), and in California for Kaiser Permanente Southern California health plan enrollees in seven counties.

**TABLE 1:** Incidence of type 1 diabetes per 100,000 persons per year and annual percent change (APC) in incidence in youths aged <20 years, overall and by age at diagnosis, sex, and race/ethnicity — SEARCH for Diabetes in Youth Study (SEARCH), United States,* 2002–2015 –

Characteristic	Incidence rate (95% CI)		Adjusted APC^§^ 2002–2010 (95% CI)	Incidence rate (95% CI)		Adjusted APC^†^ 2011–2015 (95% CI)	Adjusted APC^§^ 2002–2015 (95% CI)	Change from 2002–2010 to 2011–2015 (95% CI)
	2002–2003	2009–2010		2011–2012	2014–2015			
**Overall**	**19.5 (18.3 to 20.8)**	**20.4 (19.2 to 21.7)**	**2.0 (0.9 to 3.2)^¶^**	**21.7 (20.4 to 23.0)**	**22.3 (21.0 to 23.6)**	**1.9 (0.9 to 2.9)^¶^**	**1.93 (1.34 to 2.51)^¶^**	**−0.1 (−1.9 to 1.6)**
**Age group at diagnosis (yrs)**								
0–4	16.5 (14.3 to 19.1)	13.8 (11.9 to 16.0)	0.3 (−2.2 to 3.0)	14.3 (12.4 to 16.6)	14.4 (12.4 to 16.7)	0.8 (−1.4 to 3.0)	0.6 (−0.72 to 1.93)	0.4 (−3.5 to 4.5)
5–9	24.0 (21.3 to 27.0)	27.5 (24.7 to 30.6)	3.3 (1.2 to 5.3)^¶^	27.7 (25.0 to 30.8)	27.1 (24.4 to 30.1)	0.9 (−0.8 to 2.7)	1.91 (0.86 to 2.98)^¶^	−2.3 (−5.3 to 0.8)
10–14	26.4 (23.7 to 29.3)	28.7 (25.9 to 31.8)	1.7 (−0.2 to 3.7)	31.8 (28.8 to 35.0)	33.5 (30.5 to 36.8)	3.0 (1.3 to 4.8)^¶^	2.4 (1.39 to 3.42)^¶^	1.3 (−1.7 to 4.4)
15–19	11.0 (9.2 to 13.0)	12.0 (10.3 to 14.1)	2.7 (0.1 to 5.4)^¶^	12.9 (11.1 to 15.0)	13.6 (11.8 to 15.8)	2.3 (0.1 to 4.6)^¶^	2.44 (1.09 to 3.8)^¶^	−0.4 (−4.3 to 3.7)
**Sex**								
Female	19.2 (17.5 to 21.0)	19.7 (18.1 to 21.6)	2.5 (0.8 to 4.2)^¶^	19.9 (18.3 to 21.8)	20.4 (18.7 to 22.3)	1.0 (−0.4 to 2.4)	1.5 (0.68 to 2.33)^¶^	−1.4 (−3.9 to 1.1)
Male	19.8 (18.2 to 21.7)	21.0 (19.3 to 22.9)	1.8 (0.2 to 3.4)^¶^	23.4 (21.6 to 25.3)	24.1 (22.2 to 26.0)	2.6 (1.3 to 4.0)^¶^	2.33 (1.54 to 3.12)^¶^	0.8 (−1.6 to 3.3)
**Race/Ethnicity**								
White	23.9 (22.2 to 25.7)	25.4 (23.7 to 27.4)	1.2 (−0.1 to 2.6)	27.0 (25.2 to 29.0)	27.3 (25.5 to 29.3)	0.5 (−0.7 to 1.7)	0.73 (0.02 to 1.44)^¶^	−0.8 (−2.9 to 1.4)
Black	14.7 (12.1 to 17.7)	15.5 (12.9 to 18.6)	1.2 (−1.5 to 3.9)	19.0 (16.1 to 22.4)	20.8 (17.7 to 24.4)	4.0 (1.7 to 6.3)^¶^	2.72 (1.42 to 4.03)^¶^	2.8 (−1.4 to 7.1)
Hispanic	13.7 (11.4 to 16.4)	16.3 (14.0 to 18.9)	5.9 (3.4 to 8.6)^¶^	14.8 (12.7 to 17.3)	16.3 (14.1 to 18.8)	2.5 (0.5 to 4.6)^¶^	4.05 (2.84 to 5.28)^¶^	−3.2 (−6.7 to 0.4)
Asian/Pacific Islander	7.9 (5.0 to 12.3)	5.5 (3.4 to 8.9)	−1.5 (−7.4 to 4.8)	9.8 (6.8 to 13.9)	9.4 (6.6 to 13.3)	8.5 (3.2 to 14.0)^¶^	4.36 (1.44 to 7.37)^¶^	10.1 (0.1 to 21.1)^¶^
American Indian**	6.6 (3.5 to 12.8)	5.0 (2.3 to 10.7)	−2.0 (−12.2 to 9.5)	6.5 (3.3 to 12.9)	6.2 (3.0 to 12.9)	3.7 (−5.8 to 14.2)	1.17 (−4.05 to 6.68)	5.8 (−11.2 to 26.1)

**Abbreviation:** CI = confidence interval.

* SEARCH includes data on youths (<20 years) in Colorado (all 64 counties plus selected Indian reservations in Arizona and New Mexico under the direction of Colorado), Ohio (eight counties), South Carolina (all 46 counties), Washington (five counties), and in California for Kaiser Permanente Southern California health plan enrollees in seven counties.

^†^ APC based on model with change point at 2011 and adjusted as follows: overall results adjusted by age, sex, and race/ethnicity; results by age adjusted for sex and race/ethnicity; results by sex adjusted for age and race/ethnicity; results by race/ethnicity adjusted for age and sex.

^§^ APC based on model without a change point from 2002 to 2015 and adjusted as follows: overall results adjusted by age, sex, and race/ethnicity; results by age adjusted for sex and race/ethnicity; results by sex adjusted for age and race/ethnicity; results by race/ethnicity adjusted for age and sex.

^¶^ APC and change from 2002–2010 to 2011–2015 significantly different from zero (as indicated by 95% CI that does not include zero).

** Primarily persons from one southwestern tribe.

During 2002–2015, the incidence of type 2 diabetes increased among youths aged 10–19 years in all age, sex, and race/ethnicity groups except whites (Figure) (Table 2). During 2014–2015, type 2 diabetes incidence differed by race/ethnicity, with lowest rates observed among whites (0.77) and higher rates among American Indians (3.69), blacks (5.97), and Hispanics (6.45). In the analyses adjusted for age, sex, race/ethnicity, type 2 diabetes incidence increased at a constant rate from the period 2002–2010 to 2011–2015, with an overall APC of 4.8% per year. The steepest APC increase was among Asians and Pacific Islanders (7.7% per year) followed by Hispanics (6.5% per year), blacks (6.0% per year), and American Indians (3.7% per year).

**TABLE 2:** Incidence of type 2 diabetes per 100,000 persons per year and annual percent change (APC) in incidence in youths aged 10–19 years, overall and by age at diagnosis, sex and race/ethnicity — SEARCH for Diabetes in Youth Study (SEARCH), United States,* 2002 to 2015

Characteristic	Incidence rate (95% CI)		Adjusted APC^†^ 2002–2010 (95% CI)	Incidence rate (95% CI)		Adjusted APC^†^ 2011–2015 (95% CI)	Adjusted APC^†^ 2002–2015 (95% CI)	Change from 2002–2010 to 2011–2015 (95% CI)
	2002–2003	2009–2010		2011–2012	2014–2015			
Overall	9.0 (7.9 to 10.2)	12.2 (10.9 to 13.6)	5.1 (2.9 to 7.4)^¶^	12.5 (11.2 to 13.9)	13.8 (12.4 to 15.3)	4.6 (2.7 to 6.4)^¶^	4.81 (3.7 to 5.92)^¶^	−0.6 (−3.8 to 2.7)
**Age group at diagnosis (yrs**)								
10–14	8.0 (6.6 to 9.7)	12.0 (10.2 to 14.0)	5.2 (1.9 to 8.5)^¶^	12.1 (10.3 to 14.1)	12.4 (10.6 to 14.5)	3.9 (1.3 to 6.6)^¶^	4.57 (2.95 to 6.22)^¶^	−1.2 (−5.8 to 3.6)
15–19	10.0 (8.4 to 11.9)	12.4 (10.7 to 14.5)	5.0 (2.0 to 8.2)^¶^	12.9 (11.1 to 15.0)	15.2 (13.2 to 17.5)	5.1 (2.6 to 7.7)^¶^	5.02 (3.48 to 6.58)^¶^	0.1 (−4.3 to 4.7)
**Sex**								
Female	11.1 (9.4 to 13.1)	15.8 (13.8 to 18.2)	6.6 (3.7 to 9.7)^¶^	16.1 (14.1 to 18.5)	16.7 (14.6 to 19.1)	3.9 (1.6 to 6.4)^¶^	5.11 (3.6 to 6.64)^¶^	−2.5 (−6.5 to 1.7)
Male	7.0 (5.7 to 8.6)	8.7 (7.2 to 10.4)	3.1 (−0.3 to 6.5)	9.0 (7.5 to 10.7)	11.1 (9.4 to 13.0)	5.4 (2.6 to 8.3)^¶^	4.41 (2.69 to 6.15)^¶^	2.3 (−2.6 to 7.5)
**Race/Ethnicity**								
White	4.4 (3.4 to 5.5)	4.8 (3.8 to 6.1)	1.9 (−2.3 to 6.3)	3.9 (3.0 to 5.0)	4.5 (3.5 to 5.7)	−0.1 (−3.7 to 3.5)	0.77 (−1.35 to 2.94)	−2.0 (−8.2 to 4.6)
Black	20.0 (16.0 to 25.1)	31.0 (25.8 to 37.2)	6.3 (2.6 to 10.1)^¶^	32.5 (27.2 to 38.9)	37.8 (31.9 to 44.7)	5.8 (2.8 to 8.8)^¶^	5.97 (4.14 to 7.85)^¶^	−0.5 (−5.6 to 5.0)
Hispanic	13.3 (10.2 to 17.4)	17.2 (14.0 to 21.2)	6.3 (2.2 to 10.5)^¶^	18.4 (15.2 to 22.4)	20.9 (17.4 to 24.9)	6.6 (3.4 to 9.9)^¶^	6.45 (4.44 to 8.49)^¶^	0.3 (−5.4 to 6.3)
Asian/Pacific Islander	11.0 (6.5 to 18.7)	12.9 (8.3 to 20.1)	7.9 (−0.8 to 17.4)	12.2 (7.8 to 19.0)	11.9 (7.8 to 18.3)	7.6 (1.0 to 14.6)^¶^	7.72 (3.44 to 12.19)^¶^	−0.3 (−11.8 to 12.7)
American Indian**	22.6 (13.9 to 36.8)	30.1 (19.4 to 46.5)	5.1 (−2.1 to 12.8)	45.0 (31.1 to 65.1)	32.8 (20.8 to 51.6)	2.6 (−3.2 to 8.8)	3.69 (0.11 to 7.39)^¶^	−2.3 (−12.3 to 8.7)

**Abbreviation:** CI = confidence interval.

* SEARCH includes data on youths (<20 years) in Colorado (all 64 counties plus selected Indian reservations in Arizona and New Mexico under the direction of Colorado), Ohio (eight counties), South Carolina (all 46 counties), Washington (five counties), and in California for Kaiser Permanente Southern California health plan enrollees in seven counties.

^†^ APC based on model with change point at 2011 and adjusted as follows: overall results adjusted by age, sex, race/ethnicity; results by age adjusted for sex and race/ethnicity; results by sex adjusted for age and race/ethnicity; results by race/ethnicity adjusted for age and sex.

^§^ APC based on model without a change point from 2002–2015 and adjusted as follows: overall results adjusted by age, sex, race/ethnicity; results by age adjusted for sex and race/ethnicity; results by sex adjusted for age and race/ethnicity; results by race/ethnicity adjusted for age and sex.

^¶^ APC and change from 2002–2010 to 2011–2015 significantly different from zero (as indicated by 95% CI that does not include zero).

** Primarily persons from one southwestern tribe.

## Discussion

From 2002 to 2015, the annual incidence of both type 1 and type 2 diabetes increased at constant rates among persons aged <20 years in selected counties and Indian reservations in the United States. Rates of increase in incidence were higher for type 2 diabetes (4.8% per year) than for type 1 (1.9%). Since 2012, the rate of increase in type 2 diabetes has not changed, and has also remained constant for type 1 diabetes, except among Asians and Pacific Islanders. These findings provide indicators of the number of new cases of type 1 and type 2 diabetes among U.S. youths and identify groups with increased incidences of both type 1 and type 2 diabetes. Diabetes is a chronic disease that requires lifelong treatment and management. Better understanding of the number of new cases of diabetes among youths helps in planning for health care needs and resources.

The findings in this report are subject to at least two limitations. First, a small number of cases was ascertained across years, in subgroups by diabetes type, and especially across racial/ethnic groups, possibly leading to less precision in the annual rates. Second, these findings might not be generalizable to other populations because SEARCH was not designed to be nationally representative; it includes populations from five U.S. sites. A major strength of this study is that data come from a complete, population-based registry covering approximately a decade, including both type 1 and type 2 diabetes in persons aged <20 years across multiple racial/ethnic groups.

The incidence of type 1 diabetes continues to increase in U.S. youths, with steeper increases observed in black and Hispanic youths. Since 2011, the incidence of type 1 diabetes has also significantly increased among Asians and Pacific Islanders. Reasons for this recent increase are unknown. In parallel with increased obesity prevalence in U.S. youths (*9*), the incidence of type 2 diabetes among adolescents has increased at a higher rate than that of type 1 diabetes, especially among racial/ethnic minority youths. There are no known prevention interventions for type 1 diabetes; in adults the onset of type 2 diabetes can be prevented or delayed with lifestyle changes programs, such as the National Diabetes Prevention Program (https://www.cdc.gov/diabetes/prevention/index.html). Although the effectiveness of these programs among youths is unknown, promoting healthy eating and lifestyles provides many health benefits (https://www.cdc.gov/diabetes/prevent-type-2/type-2-kids.html). One program targeting the prevention of type 2 diabetes in American Indian youths is the Native Diabetes Wellness Program (https://www.cdc.gov/diabetes/ndwp/index.html). This collaboration between CDC and other partners provides resources to promote healthy eating and physical activity in American Indian and Alaska Native youths. To assess public health needs and prevention efforts for type 1 and type 2 diabetes among youths, it is important to enhance and continue surveillance efforts to monitor incidence in this population.

**Summary**

**What is already known about this topic?**

Diabetes, one of the most common chronic diseases among youths, is associated with numerous complications, and has a substantial impact on public health resources. From 2002 to 2012, type 1 and type 2 diabetes incidence has increased among U.S. youths aged <20 years.

**What is added by this report?**

From 2011 to 2015, both type 1 and type 2 diabetes incidence continued to increase among youths at five U.S. sites included in the SEARCH for Diabetes in Youth Study, especially among racial and ethnic minority populations.

**What are the implications for public health practice?**

Ongoing surveillance to monitor trends in type 1 and type 2 diabetes incidence can help identify population subgroups at increased risk for diabetes to aid prevention efforts and planning for future health care needs.
